# Adalimumab-induced Anti-neutrophilic Cytoplasmic Antibody Vasculitis: A Rare Complication of an Increasingly Common Treatment

**DOI:** 10.7759/cureus.5598

**Published:** 2019-09-09

**Authors:** Tanya Chandra, Joy-Ann Tabanor-Gayle, Santhanam Lakshminarayanan

**Affiliations:** 1 Internal Medicine, University of Connecticut Health Center, Farmington, USA; 2 Rheumatology, University of Connecticut Health Center, Farmington, USA

**Keywords:** tnf inhibitor, anca vasculitis, adverse drug reactions

## Abstract

Tumor necrosis factor (TNF) inhibitors are used for treatment of different autoimmune diseases. Interestingly they are also being noted to cause autoimmune side effects such as vasculitis, systemic lupus erythematosus, interstitial lung disease, etc. We describe one such case of granuloma annulare being treated with Adalimumab, who then developed pulmonary-renal syndrome form anti-neutrophilic cytoplasmic antibody (ANCA)-induced vasculitis. This was treated with steroids and immunosuppression, as well as discontinuation of the TNF inhibitor. However, he remains dependant on dialysis and immunosuppression. In this article, we review the existing literature on clinical presentation and course of TNF inhibitors-induced ANCA vasculitis. We also discuss the underlying mechanisms thought to be responsible for this.

## Introduction

Tumor necrosis factor (TNF) inhibitors are utilized for the treatment of a variety of autoimmune diseases, but have also been associated with the paradoxical emergence of autoimmune phenomena, including cutaneous vasculitis. There have also been several reported cases of systemic vasculitis following treatment with Adalimumab without mention of specific patient details or circumstances [1]. Anti-neutrophilic cytoplasmic antibody (ANCA) vasculitis has been rarely described.

## Case presentation

We present the case of a 57-year-old male with past medical history significant for coronary artery disease, hypertension and granuloma annulare (GA) who was admitted with rapid decline in renal function and shortness of breath. GA was being treated with adalimumab for the last two years. Eight months prior to admission, he was noted to have an asymptomatic elevation in his blood urea nitrogen and creatinine (Table 1) which worsened five months later. Renal ultrasound was performed which showed bilateral echogenic kidneys. He was lost to follow up and represented to his primary care provider three months later with a one-week history of epistaxis, hemoptysis, anorexia and weight loss. He was asked to report to the emergency room.

**Table 1:** Laboratory data on admission ANA: Anti-Nuclear Antibody; p-ANCA: perinuclear Anti-Neutrophilic Cytoplasmic Antibody; AI: Antibody Index; c-ANCA: cytoplasmic Anti-Neutrophilic Cytoplasmic Antibody.

Laboratory Test	Results		
	During hospitalization	Three months prior	Eight months prior
Blood urea nitrogen (7-18 mg/dl)	136	51	32
Creatinine (0.55-1.3 mg/dl)	15.89	3.4	2.6
Hemoglobin (13.5-18 gm/dl)	6.1		
ANA Titre (Negative)	1:80 (homogenous)		
p-ANCA Titre (<1:20)	1:40		
Proteinase 3 antibody (<1 AI)	<1		
Myeloperoxidase antibody (<1AI)	5		
c-ANCA (Negative)	Negative		
C3 complement (82-185 mg/dl)	130		
C4 complement (15-35 mg/dl)	34		
Hepatitis B surface antigen (Negative)	Negative		
Hepatitis C virus antibody (Negative)	Negative		

**Figure 1:**
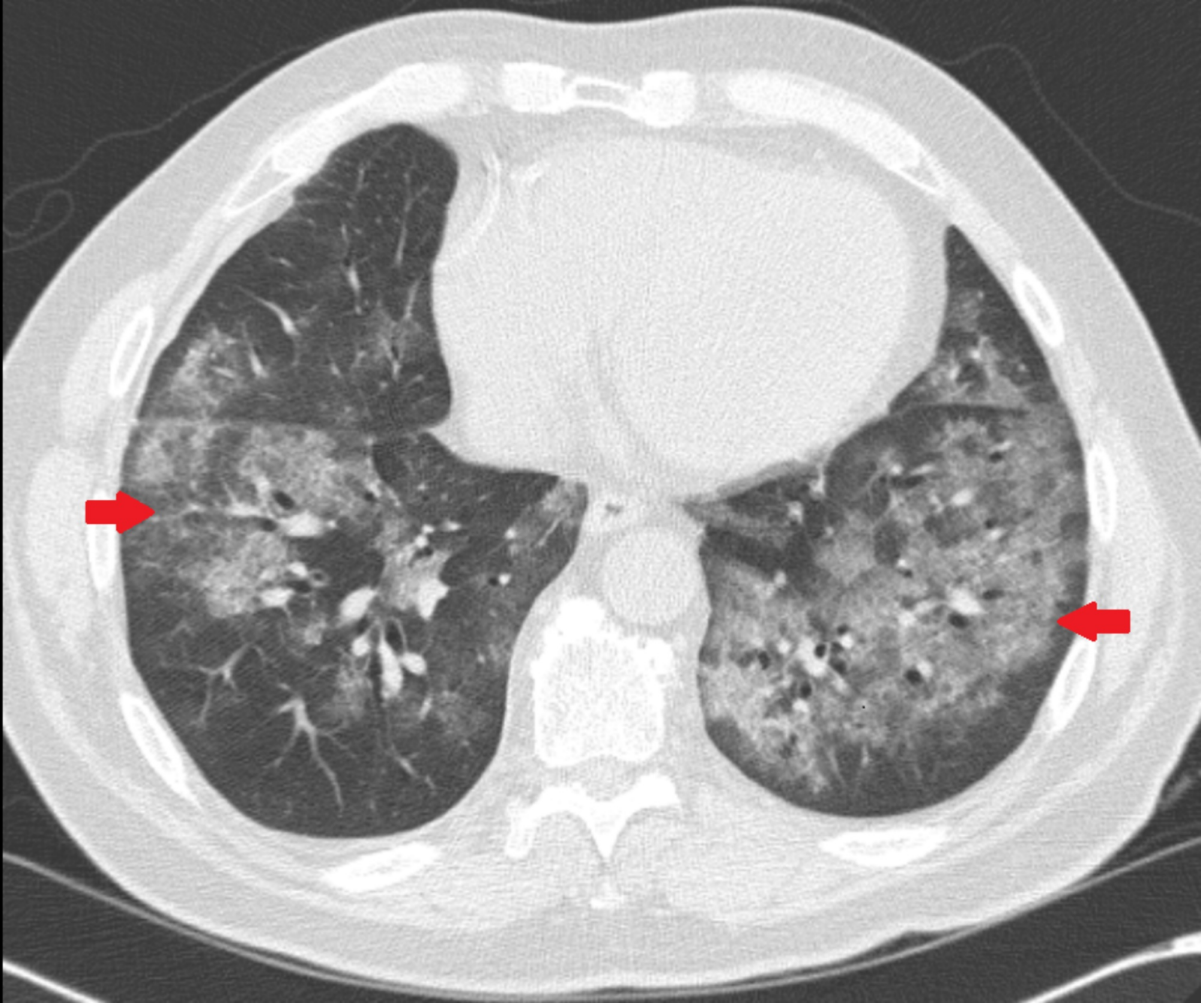
Axial CT chest image showing bilateral diffuse opacities (arrows)

**Figure 2:**
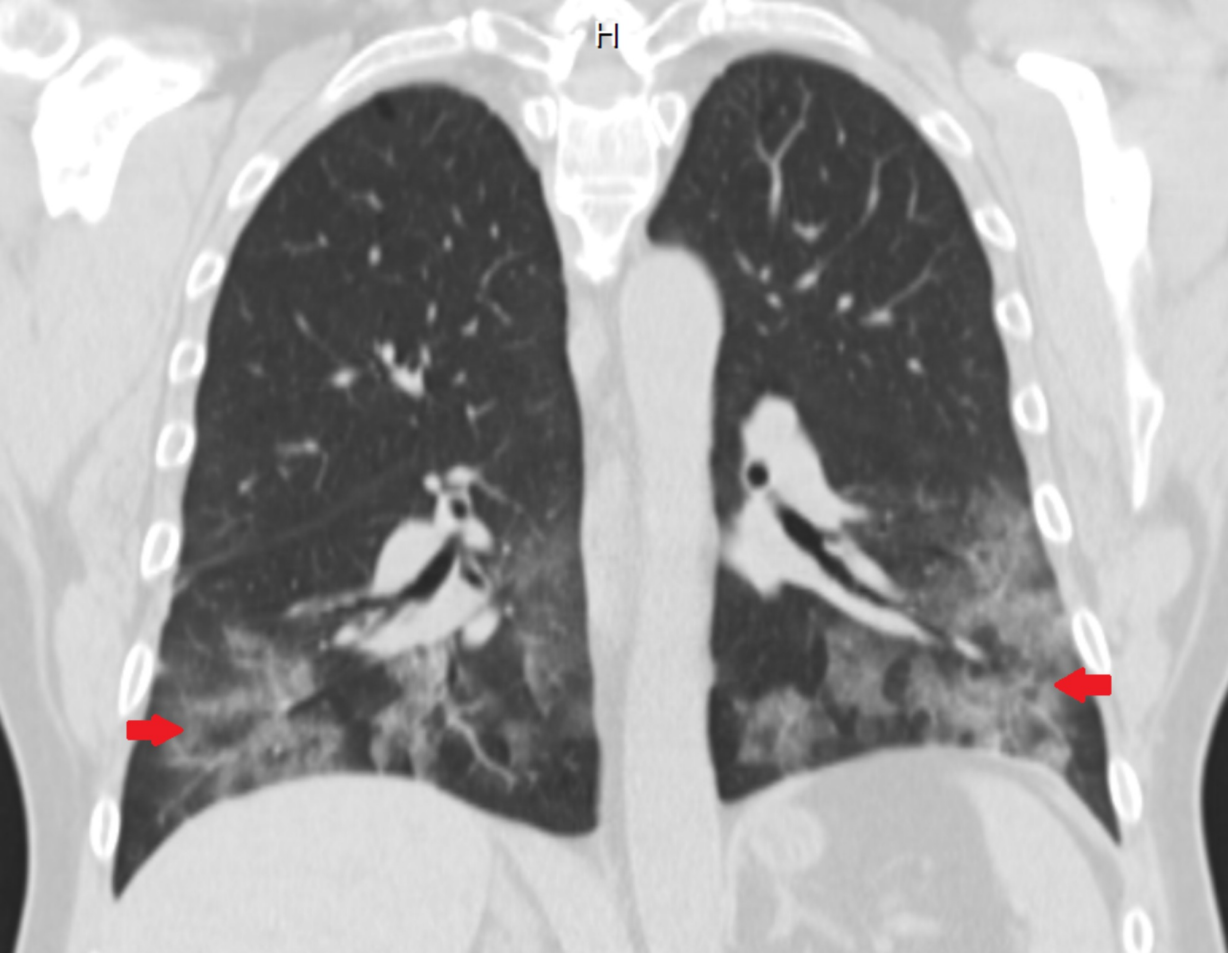
Coronal CT chest image showing bilateral basilar opacities (arrows)

**Figure 3:**
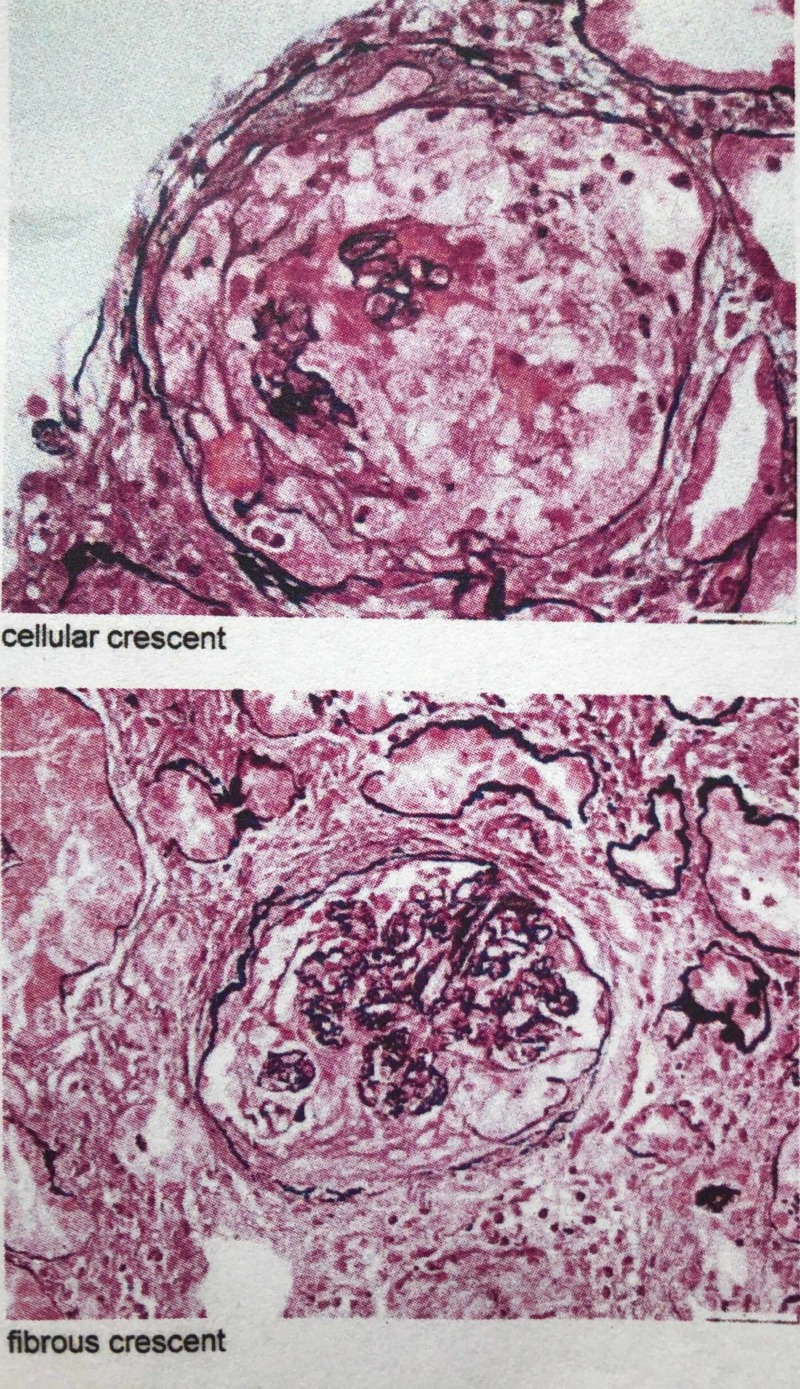
Renal biopsy images showing crescentic glomerulonephritis

CT chest showed bilateral pulmonary consolidation and ground glass opacities (Figures 1, 2). Renal biopsy performed revealed pauci-immune, rapidly progressive glomerulonephritis with some fibrosis (Figure 3). ANCA with perinuclear staining and myeloperoxidase antibody were positive. He was started on hemodialysis immediately. He also received intravenous methylprednisolone 500 mg daily for three days followed by oral prednisone 60 mg daily, oral cyclophosphamide 125 mg daily (which was eventually transitioned to intravenous monthly pulses of cyclophosphamide) and trimethoprim-sulfamethoxazole for pneumocystis prophylaxis. Adalimumab was discontinued.

Two months after his hospitalization, pulmonary infiltrates have resolved, but there has been no recovery of renal function.

## Discussion

To our knowledge, there have only been nine previously reported cases of vasculitis and positive ANCA that were thought to be induced by TNF inhibitors [2-9]. See Table 2 for clinical presentation and treatment of each patient. One patient had atypical ANCA and lupus nephritis (Patient 8) and one patient had aortitis (Patient 9) which are not consistent with true ANCA-associated vasculitis. Of the remaining seven patients, four patients were females and six were being treated for rheumatoid arthritis. The mean age for patients was 51.4 years. Time of onset of symptoms after starting a TNF inhibitor varied from three months to four years. Four of these patients had positive c-ANCA, three had a positive p-ANCA. Six of seven patients had renal biopsies showing pauci-immune glomerulonephritis. Six patients were treated with intravenous methylprednisolone followed by oral prednisone. The TNF inhibitor was discontinued in all cases except patient 7. The most commonly used immunosuppressant was cyclophosphamide in six of seven patients. Four patients had persistent renal dysfunction and one patient died within nine months of presentation. Given the temporal sequence of events, a causal relationship might be present. One proposed mechanism is that anti-TNF drugs form immune complexes, activate complement and promote switching from a T-helper type 1 response (mediated by interleukin (IL)-1, TNF and interferon (IFN)-Y) to a T-helper type 2 response (IL-4, IL-5, IL-6, IL-10 and IL-13) leading to the production of autoantibodies [10].

**Table 2:** Vasculitis with positive ANCA induced by TNF- inhibitors ANCA: Anti-Neutrophilic Cytoplasmic Antibody; TNF-i: Tumor Necrosis Factor inhibitor; CD: Crohn’s Disease; GN: Glomerulonephritis; Hb: Hemoglobin; CRP: C-Reactive Protein; RBC: Red Blood Cell; PR-3: Proteinase-3; IV: Intravenous; MP: Methylprednisolone; RA: Rheumatoid Arthritis; UPC: Urine Protein Creatinine; ANA: Anti-Nuclear Antibody; dsDNA: double stranded Deoxyribonucleic Acid; anti-GBM: anti-Glomerular Basement Membrane; HCQ: Hydroxychloroquine; MTX: Methotrexate; HD: Hemodialysis; ESR: Erythrocyte Sedimentation Rate; RF: Rheumatoid factor; MPO: Myeloperoxidase; SS: Sjogren’s Syndrome; CrCl: Creatinine Clearance; PO: Per Oral; TMP: Trimethoprim; AZA: Azathioprine; RTX: Rituximab; Cr: Creatinine.

Patient No.	Age/Sex	TNF-i	Indication for TNF-i	Time of onset after starting TNF-i (months)	Clinical presentation	Labs	ANCA type	Other serologies	Pathology	Previous/Concomitant drugs	Treatment	Outcome
	54/M	Adalimumab	CD	30	Fever, asthenia, lower extremity edema, inflammatory arthritis, polyneuropathy and optic neuritis, anemia, GN	Hb: 9 gm/dl, CRP: 7.9 mg/dl, Urine studies: 1.2 gm protein/day >50 RBCs/hpf Granular casts	C-ANCA PR3	-	Pauci-immune extracapillary GN (Kidney)	-	IV MP, IV CYC	Persistent renal dysfunction C-ANCA negative
	62/F	Adalimumab	RA	48	Malaise, weight loss nasal stuffiness, visual blurring, rash, GN	Urine studies: 3+ blood 3+ protein UPC 5.9 g	C-ANCA PR3	(+) ANA1:640 (-) dsDNA (-) anti-GBM (-) anti- Cardiolipin, Normal complements	Pauci-immune mild segmental sclerosis with no tubuloreticular lesions (Kidney)	HCQ, Sulfasalazine, MTX	IVMP, Plasma exchange, 1 HD PO prednisone, CYC	Improved UPC Persistent renal dysfunction Improved C-ANCA
	67/F	Etanercept	RA	3	Painful, erythematous ulcerated nodules, nasal congestion, peripheral neuropathy, polyarthritis, scleritis, GN pulmonary parenchymal nodules, chronic sinusitis on CT	Hb 13 gm/dl, ESR 111 mm/hr, CRP 15.3 mg/dl, Urine Studies: Hematuria	C-ANCA	(+) RF (45 IU/ml) (+) ANA 1:320 homogenous	Leukocytoclastic (Skin)	MTX, Prednisolone	IVMP pulses, CYC 750/month Steroid taper	Good clinical response
	33/F	Infliximab	RA	16	Synovitis anemia GN	Hb 8.8 gm/dl, Cr 0.6 mg/dl (CrCl 82.5 ml/min), ESR 56 mm/hr, CRP 2.5 mg/dl, Urine Studies: 3+ protein 3+ occult blood, 24 hr urine protein: 1.2 gm/day	MPO PR3	(-) Anti-DNA (-) Anti-GBM Normal IgG, IgA, IgM Normal complement	IgM deposition (weak intensity) IgG, IgA, C3, C1q and kappa and Lambda chains (-)- Necrotizing GN (Kidney)	MTX, Sulfasalazine, Bucillamine, Cyclosporine	IVMP, PO prednisone	Good clinical response
	31/M	Infliximab	RA	8	Synovitis rash GN	Cr 3.4 mg/dl (CrCl 54 ml/min), CRP 9.1 mg/dl, Urine Studies: 3+ blood 24 hr Urine protein 1.5 gm	C-ANCA PR3	(+) ANA 1:320 (homogenous) (-) dsDNA (+) RF (-) HepB and C serology (-) Cryoglobulin Normal complement	Pauci-immune crescentic GN (Kidney), Non diagnostic (Skin)	MTX, Cyclosporine Sulfasalazine, HCQ leflunomide	TMP, 1 gm IVMP for 3 days, Oral CYC 2 mg/kg daily. AZA	Good clinical response Decreased PR3
	58/F	Adalimumab	RA	48	Asymptomatic rapidly progressive GN Alveolar hemorrhage with pulmonary biopsy showing pauci-immune vasculitis anemia	Hb 6.2 gm/dl, CRP < 10 mg/dl, Urine Studies: RBCs+, 2.47 gm spot urine protein	P-ANCA MPO	(-) GBM (-) dsDNA (+) RF (+) ANA 1:640 homogeneous (+) SS-A & SS-B	Pauci-immune necrotizing GN-extracapillary necrotizing GN (Kidney)	D-penicillamine, Gold, MTX, steroids	IVMP, PO prednisone, Plasma exchanges-7 over 2 weeks, IV CYC six courses HD, AZA	Persistent renal dysfunction
	55/M	Etanercept	RA	4	Alopecia maculopapular rash lower extremity sensory neuropathy GN	Cr. 3 mg/dl, Urine Studies: 1+ protein >5 RBCs/hpf 5 WBCs/hpf no casts 24 hr urinary protein - 1 gm/day	P-ANCA	(+) ANA 1:320 (-) anti-dsDNA Normal C3 and C4	Pauci-immune focal, segmental, necrotizing and crescentic GN (Kidney)	MTX	IV CYC	Died
	52/F	Adalimumab	RA	3	Gross hematuria and acute renal failure, GN	Urine Studies: 3+ protein >20 RBC/hpf granular casts 3.8 gm proteinuria	Atypical ANCA	(+) RF (+) ANA 1:640 homogenous, (+) dsDNA 1:25 IgG (-) cryoglobulin Decreased C3 and C4	Focal proliferative lupus nephritis (class 3) (Kidney)	Prednisone, MMF, Infliximab, MTX, HCQ Penicillamine, Gold	Pulse IVMP PO steroids for 1 month	Persistent renal dysfunction
	65/F	Etanercept	AS	36	Worsening cervical pain Severe and extensive aortitis on CTA chest and abdomen	Hb 9 mg/dl, ESR > 100 mm/hr, CRP 23.9 mg/dl	C-ANCA MPO	Borderline ANA (-) RF Normal complements			IVMP 1 g for 3 days, RTX, Prednisone	Good clinical response

## Conclusions

TNF-induced ANCA vasculitis is exceedingly rare. These cases in conjunction with ours suggest a possible relationship between anti-TNF use and induction of ANCA vasculitis. To the best of our knowledge, our case is the 4th to describe TNF-induced pulmonary renal syndrome as a manifestation of ANCA-vasculitis. It is difficult to conclusively prove that it is not just a spontaneous emergence of ANCA in a patient predisposed to autoimmunity. However, the biological plausibility of shifting towards a T-helper 2 type response in a susceptible individual leading to the emergence of these antibodies among others, remains. Additionally, this subset of patients appears to have a predilection for rapidly progressive kidney injury with long-term impairment despite discontinuation of the anti-TNF agent. This highlights the need for further studies looking into recognizing risk factors for the development of this rare but significant complication.
